# Increased resting state connectivity in the anterior default mode network of idiopathic epileptic dogs

**DOI:** 10.1038/s41598-021-03349-x

**Published:** 2021-12-13

**Authors:** Katrin M. Beckmann, Adriano Wang-Leandro, Henning Richter, Rima N. Bektas, Frank Steffen, Matthias Dennler, Ines Carrera, Sven Haller

**Affiliations:** 1 Section of Neurology, Department of Small Animals, Vetsuisse Faculty Zurich, University of Zurich, Zurich, Switzerland; 2 Clinic for Diagnostic Imaging, Department of Diagnostics and Clinical Services, Vetsuisse-Faculty Zurich, University of Zurich, Zurich, Switzerland; 3 Clinic for Neuroradiology, University Hospital Bonn, Bonn, Germany; 4 Section of Anaesthesiology, Department of Diagnostics and Clinical Services, Vetsuisse Faculty, University of Zurich, Zurich, Switzerland; 5 Willows Veterinary Centre and Referral Service, Highlands Road, Shirley, UK; 6 Department of Surgical Sciences, Radiology, Uppsala University, Uppsala, Sweden; 7 Faculty of Medicine, University of Geneva, Geneva, Switzerland

**Keywords:** Epilepsy, Network models

## Abstract

Epilepsy is one of the most common chronic, neurological diseases in humans and dogs and considered to be a network disease. In human epilepsy altered functional connectivity in different large-scale networks have been identified with functional resting state magnetic resonance imaging. Since large-scale resting state networks have been consistently identified in anesthetised dogs’ application of this technique became promising in canine epilepsy research. The aim of the present study was to investigate differences in large-scale resting state networks in epileptic dogs compared to healthy controls. Our hypothesis was, that large-scale networks differ between epileptic dogs and healthy control dogs. A group of 17 dogs (Border Collies and Greater Swiss Mountain Dogs) with idiopathic epilepsy was compared to 20 healthy control dogs under a standardized sevoflurane anaesthesia protocol. Group level independent component analysis with dimensionality of 20 components, dual regression and two-sample *t* test were performed and revealed significantly increased functional connectivity in the anterior default mode network of idiopathic epileptic dogs compared to healthy control dogs (p = 0.00060). This group level differences between epileptic dogs and healthy control dogs identified using a rather simple data driven approach could serve as a starting point for more advanced resting state network analysis in epileptic dogs.

## Introduction

Epilepsy represents one of the most common chronic neurological diseases in dogs and humans, affecting approximately 0.64% of the human^1^ and 0.6–0.75% of the canine population^2,3^. Epilepsy is considered a disease of the brain network^4^. Resting state functional magnetic resonance imaging (rs-fMRI) is a non-invasive tool to evaluate network structure in neurological diseases such as epilepsy. It can be performed in non-task related conditions and during anaesthesia^5^. Over the last decade, rs-fMRI has become increasingly important as a research tool for understanding brain networking during epileptic syndromes^6–8^. Resting state networks (RSN) share similar features in humans and animals^9^ but information regarding rs-fMRI in epilepsy in non-human species are spare and limited to rodent models so far^7^. Although the dog is an established large animal model for human epilepsy^10,11^, studies describing rs-fMRI of the canine brain in dogs affected by naturally-occurring epilepsy are currently lacking.

In human epileptic syndromes, rs-fMRI has shown altered functional connectivity in different large-scale networks including attentional^12^, perceptual^13^ and default mode^5,14,15^. These alterations of large-scale networks potentially explain seizure generation and spread and therefore give insight into the pathophysiology of epilepsy^7^. The most widely studied network in patients affected by epilepsy is the default mode network (DMN)^7^. The alteration detected depend on the underlying epileptic syndrome and therefore could potentially be used as a diagnostic and prognostic biomarker^16^. Epilepsy is commonly accompanied by neurobehavioral and/or psychiatric comorbidities such as depression, anxiety and cognitive impairments^17,18^. Emerging evidence suggests that network alterations detected by rs-fMRI do not only correlate with generation of ictal activity, but may also contribute to neurobehavioral comorbidities^19^.

Naturally occurring canine idiopathic epilepsy has been proposed as a translational model for human epilepsy because the two species share similarities such as prevalence, clinical manifestation, electroencephalographic manifestation, and pharmacological response^10,11,20–23^. However, characterization of brain regions involved in canine epilepsy is lacking^11,21,23,24^. For example, the extent to which the temporal lobe is involved remains controversial^25^. In dogs, pedigree studies have demonstrated an hereditary basis for idiopathic epilepsy in several breeds including Border Collies^26,27^ and greater Swiss mountain dogs^28^. Furthermore, neurobehavioral comorbidities reported in human epilepsy, such as anxiety disorder^29^, attention-deficit/hyperactivity disorder^30,31^ and cognitive impairment^32,33^, have been recently recognized in canine epilepsy and have been reported to negatively impact the quality of life of the effected dogs^34,35^. Therefore, privately-owned epileptic dogs make it possible to bridge the gap between experimental animal models and a realistic clinical setting.

Magnetic resonance imaging of the brain is performed routinely as part of the clinical work-up of the canine idiopathic epileptic patient^36^. Currently, clinical imaging is mainly limited to the morphological structure of the brain; however, increased availability of high-field scanners in veterinary medicine have raised the demand for advanced imaging techniques^37^. Moreover, feasibility and consistency of rs-fMRI in awake and anesthetized dogs have been demonstrated recently, which opens the door for translational research^38–40^. It is particularly interesting that detected canine networks include those with altered resting state connectivity in human epilepsy. While in human epilepsy pattern of altered connectivity is depended on the underlying epileptic syndrome, lack of standardization of epileptic syndromes and lack of characterization of brain regions involved in canine epilepsy does not allow precise assumptions regarding possible localization of altered connectivity in canine epilepsy beforehand^11,21,23,24^. In human epilepsy commonly areas of decreased connectivity possibly alongside with areas of increased connectivity can be identified^41^. Therefore, we also expected areas of decreased connectivity possibly alongside with areas of increased connectivity in our study without being able to predict specific areas.

The aim of our study was to investigate differences in large-scale RSN in epileptic dogs compared to healthy controls. Our hypothesis was, that large-scale networks differ between epileptic dogs and healthy control dogs.

## Material and methods

### Subjects

Border Collies and greater Swiss Mountain dogs diagnosed with idiopathic epilepsy according to the veterinary epilepsy task force criteria^42^ and breed-matched healthy control dogs of were prospectively enrolled in this study during a period of 3.2 years (2017–2020). Additionally, 10 research beagles were included in the healthy control group. These beagle dogs have been part of a preliminary study investigating the feasibility of RSN detection under general anaesthesia^39^.

The present study is in accordance with the Swiss Animal Welfare Act (TSchG, 2005) and the Swiss Animal Welfare Ordinance (TSchV, 2008) and was approved by the Swiss Federal Veterinary Office Zurich (animal license numbers ZH272/16 and ZH046/20) ethics committee. The authors complied with the ARRIVE guidelines.

All dogs underwent clinical and neurological examinations by a board-certified veterinary neurologist. Pre-anesthetic laboratory as recommend by the international veterinary epilepsy task force for investigations of idiopathic epilepsy was performed including a complete blood cell count and serum biochemistry panel, electrolytes as well as fasted ammonia, bile acids, and urinalysis^42^. MRI of the brain including structural imaging and fMRI was performed in all dogs. Afterwards, cerebrospinal fluid was collected from the cisterna cerebellomedullaris in idiopathic epileptic dogs; nucleated cell count, differential cell count and total protein concentration were evaluated.

Dogs that fulfilled the TIERII criteria for canine idiopathic epilepsy (suspected genetic epilepsy) based on the criteria of the veterinary epilepsy task force^42^ and manifested generalized tonic–clonic seizures built the group of the idiopathic epileptic dogs.

The group of healthy control dogs included relatives of the epileptic dogs (Border collies and greater Swiss mountain dogs) and a group of healthy beagle dogs from the above-mentioned preliminary study. All these dogs without history of seizures had to have a normal clinical and neurological examination. Further inclusion criteria represented a normal hematologic and blood-chemical work-up and a normal structural MRI examination of the brain (structural MRI reported as normal by a board-certified (ECVDI) radiologist).

Animal preparation and rs-fMRI protocol were performed as previously described^39^:

Shortly, Butorphanol served as premedication. (0.1–0.2 mg/kg). Depending on the temperament of the dog Butorphanol was administered intramuscularly prior to catheter placement or intravenously after catheter placement. Oro-tracheal intubation was performed, after induction of anaesthesia with Propofol 1% (MCT Fresenius Kabi, Oberdorf, Switzerland) IV, given to effect. Anaesthesia was maintained with sevoflurane vaporized in oxygen and medical air. Vaporizer settings were adjusted to the minimum possible dosage to prevent motion of the dogs. End-tidal concentration of sevoflurane, heart rate (HR), respiratory rate (RR), non-invasive mean blood pressure, percutaneous arterial oxygen saturation (SpO_2_) and end-tidal partial pressure of carbon dioxide (EtCO_2_) were monitored using a medical monitor (DatexOhmeda) and recorded every 5 min. Normocapnia (EtCO_2_ between 35 and 38 mmHg) was maintained and FiO_2_ was kept between 45–61%. Blood pressures were maintained stable within physiological values under general anaesthesia (Mean arterial blood pressure (MAP) > 60 mmHg) using RiAc infusion.

MAP under 60 mmHg was treated first with fluid/RiAc challenges of 3 ml/kg IV administered over 5–10 min. After 3 fluid challenges of RiAc, a dobutamine (Dobutrex, Teva Pharma AG, Basel, Switzerland) constant rate infusion (1-5 μg/kg/min) was started to maintain MAP over 60 mmHg.

### Image acquisition

All data were acquired as previously described^39^ with a 3 Tesla scanner (Philips Ingenia, Philips AG, Zurich, Switzerland) using a 16‐channel receive‐transmit head coil (dStream HeadSpine coil solution, Philips AG). From the anatomical evaluation, a 3D T1-weighted (T1W; TR 13 ms; TE 6 ms; FOV 130 mm; slice thickness 0.6 mm; Flip angle 8°) sequence was depicted for the registration with anatomical images. Following the anatomical scans, around one hour after induction of anaesthesia, rs-fMRI scans were acquired in all dogs.

A gradient-echo planar imaging (EPI) sequence was performed using the following protocol: TR 2.0 s; TE 30 ms; FOV 236 mm; slice thickness 3.0 mm; acquisition time 12.07 min. Phase encoding direction was anterior–posterior.

### MR data processing

Processing of the rs-fMRI data was carried out using FEAT (FMRI Expert Analysis Tool) Version 6.04, part of FSL (FMRIB's Software Library, http://www.fmrib.ox.ac.uk/fsl). The following pre-statistics processing was applied: motion correction using MCFLIRT^43^, non-brain removal using BET^44^, spatial smoothing using a Gaussian kernel of FWHM 5 mm; grand-mean intensity normalization of the entire 4D dataset by a single multiplicative factor and high-pass temporal filtering (Gaussian-weighted least-squares straight line fitting, with sigma = 50.0 s). Registration to high resolution structural and standard space images was carried out using FLIRT^43,45^. As standard space image, a recently published, open-source stereotactic atlas of the canine brain was used^46^. Registration from high resolution structural to standard space was then further refined using FNIRT nonlinear registration^47,48^ Degree of motion of each dog as well as the quality of registration were assessed afterwards.

### Independent component analysis

Due to different number of healthy control dogs and epileptic dogs from each breed, independent component analysis (ICA) was first performed only in the healthy control group and the RSN were compared across breeds to assess possible effects of breed on RSNs. A second independent component analysis was performed in all healthy control and epileptic dogs together to identify differences between healthy controls and epileptic dogs.

Analysis was carried out using Probabilistic Independent Component Analysis^49^ as implemented in MELODIC (Multivariate Exploratory Linear Decomposition into Independent Components) Version 3.15, part of FSL (FMRIB’s Software Library, http://www.fmrib.ox.ac.uk/fsl). The following data pre-processing was applied to the input data: masking of non-brain voxels; voxel-wise de-meaning of the data and normalisation of the voxel-wise variance. Pre-processed data were whitened and projected into a 20-dimensional subspace using Principal Component Analysis. Twenty components were chosen based on the results of our previous study^39^. The whitened observations were decomposed into sets of vectors which describe signal variation across the temporal domain (time-courses), the session/subject domain and across the spatial domain (maps) by optimising for non-Gaussian spatial source distributions using a fixed-point iteration technique^50^. Estimated component maps were divided by the standard deviation of the residual noise and the threshold set by fitting a mixture model to the histogram of intensity values^51^.

Criteria for selection of RSNs in the different components were the following: (1) consistency of relatively large continuous regions of increased BOLD signal, (2) predominant bilaterality and/or (3) reference to anatomical landmarks or previously described RSNs in existing literature of dogs^40,52–56^. The detected RSNs were overlaid with the mean functional image of all dogs and with the selected RSNs and were visually assessed to rule out distortion and signal loss from susceptibility artefacts^38^. Furthermore signal to noise ratio maps were generated to evaluate the influence of differences in signal to noise ratios between both groups (Supplementary Materials M1).

### Statistical analysis

Descriptive statistics and non-parametric variance analysis by means of Mann–Whitney test were performed to compare population characteristics and physiological parameters during anaesthesia between both groups with a significance threshold of p < 0.05.

For fMRI data, mean deviation time series of six affine parameters (three translations and three rotations) were plotted to evaluate whether there was significant head movement during the functional scan. Furthermore, one and two sample *t* test after dual regression analysis of the data were performed. Specifically, a set of spatial maps from the group-average analysis was used to generate subject-specific versions of the spatial maps, and associated time series, using dual regression^57^. First, for each subject, the group-average set of spatial maps was regressed (as spatial regressors in a multiple regression) into the subject's 4D space–time dataset. This resulted in a set of subject-specific time series, one per group-level spatial map. Afterwards, those time series were regressed (as temporal regressors, again in a multiple regression) into the same 4D dataset, resulting in a set of subject-specific spatial maps, one per group-level spatial map. A one sample *t* test was performed using FSL's randomize permutation-testing tool^58^. Clusters of increased or decreased BOLD signal within the RSNs were confirmed using a significance level of p < 0.05. Furthermore, two sample *t* test was performed for between group comparison of the individual components in both directions. For the between group comparison p-values were corrected via Bonferroni for the number of independent components defined and for running the test in both directions: for significant changes between the groups p < 0.003125 had to be reached for epileptic versus healthy control dogs. A second two sample *t* test was performed for the signal to noise ratio maps between both groups in both directions (Supplementary Material M1 and Supplementary Fig. S4).

## Results

### Study population

Twenty Border Collies, seventeen Greater Swiss Mountain Dogs and ten research Beagles matched the inclusion criteria and were initially included in the study.

Three Border Collies and two Greater Swiss Mountain Dogs were excluded due to acquisition problems of raw data. Two Border Collies and three Greater Swiss Mountain Dogs were excluded because of insufficient quality of the registration of the functional images to the high-resolution images. The summary registration fMRI to standard space of all 37 included dogs can be found in the Supplementary Materials (Fig. S1).

For the ICA 15 Border Collies, 12 Greater Swiss Mountain Dogs and 10 Beagle dogs were included. Of those 37 dogs included in the ICA, 17 dogs suffered from idiopathic epilepsy (11 Border Collies and 6 Greater Swiss Mountain Dogs) and 20 dogs served as healthy controls (4 Border Collies, 6 Greater Swiss Mountain Dogs and 10 Beagles). All greater swiss mountain dogs and all but one Border collie of the healthy control dogs had either one littermate or one direct offspring with epilepsy. The remaining border collie had an undefined familiar history of epilepsy. Thirteen dogs were females (9 sexually intact, 4 spayed) and 24 were males (20 sexually intact, 4 castrated). Age ranged from 0.8–8.5 years (mean age 4.7 years). The body weight was between 9.6 and 70 kg (mean weight 28.9 kg). The population characteristics of both groups is summarized in Table 1. Both subpopulations showed no significant difference related to age, weight, RR, HR, SEVO_begin_ BOLD and SEVO_end_ BOLD (p > 0.05, p_min_ = 0.053, p_max_ = 0.220). Time interval from the last reported seizure to MRI ranged from 2 days to 1 month. Details about seizure semiology are summarized in Table 2.

**Table 1:** Population characteristics.

	Epileptic dogs	Healthy controls
**Breed**		
Beagle	0	10
Border Collie	11	4
Greater Swiss Mountain Dog	6	6
**Sex**		
Male	10	9
Male castrated	1	3
Female	2	8
Female spayed	4	0
Ratio male:female	11:6	3:2
**Bodyweight**		
kg [median, range]	32.6, 14.0–70.0	25.7, 9.6–52.0
**Age**		
Years [median, range]	3.7, 0.8–8.5	5.0, 1.2–8.5

**Table 2:** Semiology of epileptic events in the affected population.

	[n]
**Medical treatment at the timepoint of MRI**	
Phenobarbital	9
Potassium bromide	4
Levetiracetam	3
Imepitoin	1
Type of therapy	
Mono	2
Double	5
Triple	2
**Special diet or dietary supplement before MRI**	
Medium chain triglycerides	2
Cannabidiol	2
**Time between first seizure to MRI**	
< 1 month	2
> 1–3 months	6
> 3–12 months	6
> 12 months	3
**Time between last reported seizure to MRI**	
2–7 days	6
8–31 days	11
**Seizures**	
Status epilepticus	1
Cluster seizures	6
Seizure semiology	
Tonic–clonic	16
Tonic	1
Focal onset secondary generalisation	9
Unknown onset	8
Additional focal seizures	1
Autonomic signs	
Salivation	9
Urination	11
Defecation	5
Postictal aggression	4
**Interictal behavioural changes**	
Anxiety	3

### Anaesthesia

Anaesthesia was uneventful in all dogs. End-tidal Sevofluran level during rs-fMRI ranged from 1.8 to 3.8% (mean: 2.4%) in all dogs. Compared to the healthy control group, mean end-tidal sevoflurane was slightly higher in the epileptic dogs. It ranged from 1.8 to 2.9% (mean: 2.6%) in the healthy control dogs and from 1.9 to 3.8% (mean: 2.3%) in the epileptic dogs. Heart rate varied during rs-fMRI between 48 to 130/min (mean: 81/min). The mean heart rate in epileptic dogs was 78 beats/min (range from 48 to 105 beats/min), slightly lower than in healthy controls (mean: 88 beats/min, range from 58 to 130 beats/min).

### Motion correction

The motion was minimal in all patients with a mean relative displacement of 0.08 mm (0.03–0.32 mm) and a mean absolute displacement 0.08 mm (0.04–0.21 mm).

### Results independent component- and dual-regression-analysis

**Figure 1:**
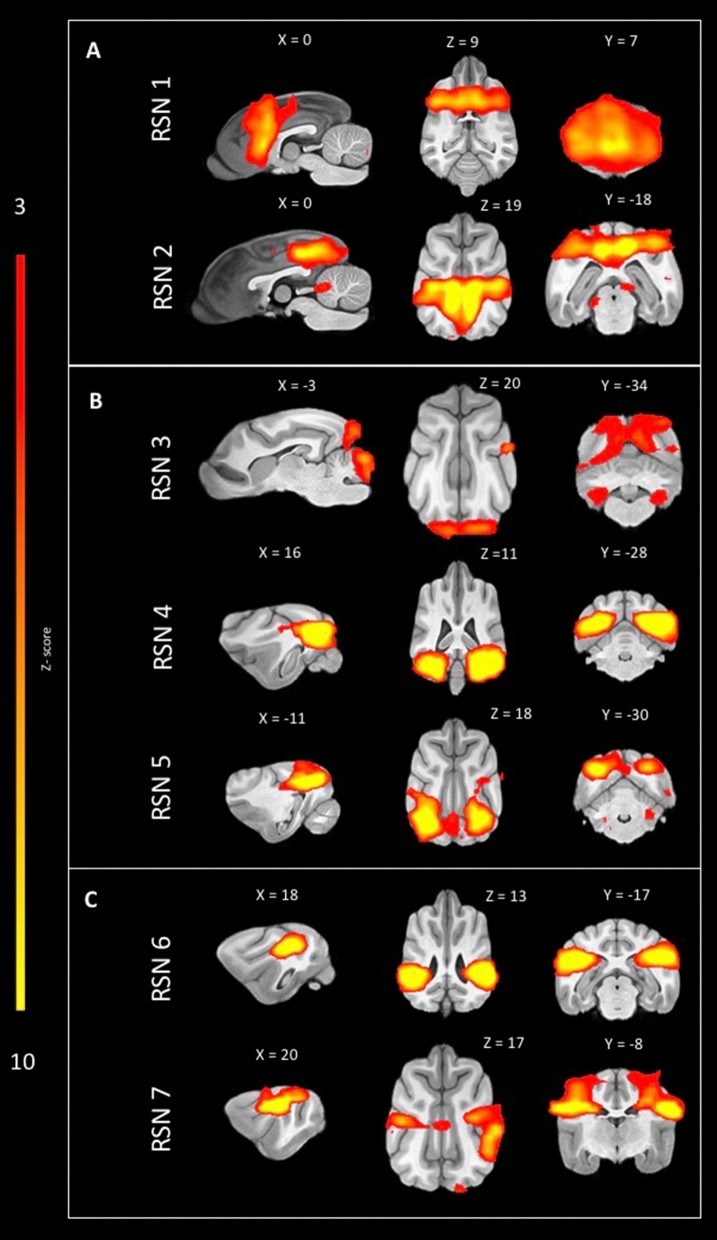
Sagittal, dorsal and transversal images of RSN obtained by means of group ICA of the healthy control dogs only and overlaid on a T1W open-source stereotactic atlas^46^. The results are reported as local false discovery rate controlled (p < 0.05) thresholded z-maps, with red-yellow color encoding using a 3 < Z-score threshold. This figure was created using FSLeyes (version 2.1 https://fsl.fmrib.ox.ac.uk/fsl/fslwiki/FSLeyes) and Microsoft Powerpoint (version 16.16.19, http://www.microsoft.com).

In the control population, seven RSNs were identified and labelled according to Uddin et al.^59^. Initially, the identified RSN were labelled according to their anatomical localisation, and in a second step a cognitive label was given if possible^59^. One network was identified as medial frontoparietal network (Fig. 1A: RSN 1 anterior DMN), one was identified as a limbic-occipital network (Fig. 1A: RSN2 posterior DMN)**,** three networks were identified in the occipital cortex (Fig. 1B: RSN3: primary visual, RSN 4 and 5 higher order visual) and two networks were identified in areas corresponding to the pericentral networks (Fig. 1C: RSN 6 and RSN 7: auditory).

After dual regression analysis, no significant differences were detected in RSNs when comparing the different breeds, therefore excluding the effect of breed on RSNs and indicating that the observed networks can be compared across breeds.

**Figure 2:**
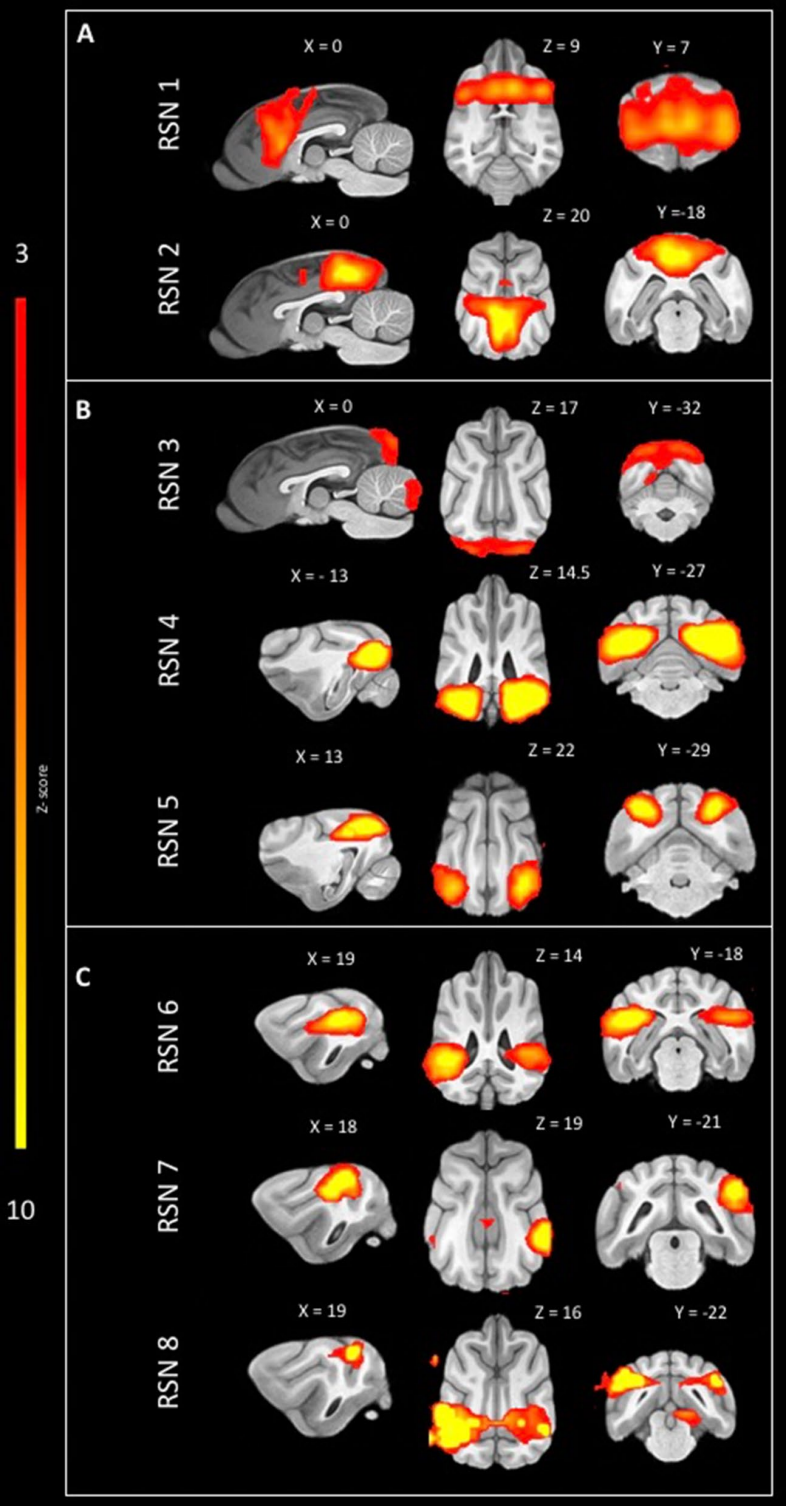
Sagittal, dorsal and transversal images of RSN obtained by means of group ICA of healthy control and epileptic dogs overlaid on a T1W open-source stereotactic atlas^46^. The results are reported as local false discovery rate controlled (p < 0.05) thresholded z-maps, with red-yellow color encoding using a 3 < Z-score threshold. This figure was created using FSLeyes (version 2.1 https://fsl.fmrib.ox.ac.uk/fsl/fslwiki/FSLeyes) and Microsoft Powerpoint (version 16.16.19, http://www.microsoft.com).

When comparing ICA between the population of epileptic dogs with controls (Fig. 2), a total of eight RSNs were identified and labelled as described above. Of those eight networks, one was identified as medial frontoparietal network (Fig. 2A: RSN 1 anterior DMN), one was identified as limbic-occipital network (Fig. 2A: RSN 2 posterior DMN)**,** three were identified in the occipital cortex (Fig. 2B: RSN3: primary visual, RSN 4 and RSN 5: higher order visual) and three were identified in areas corresponding to the pericentral networks (Fig. 2C: RSN 5 and RSN 6: auditory and RSN 7 somatosensory network).

**Figure 3:**
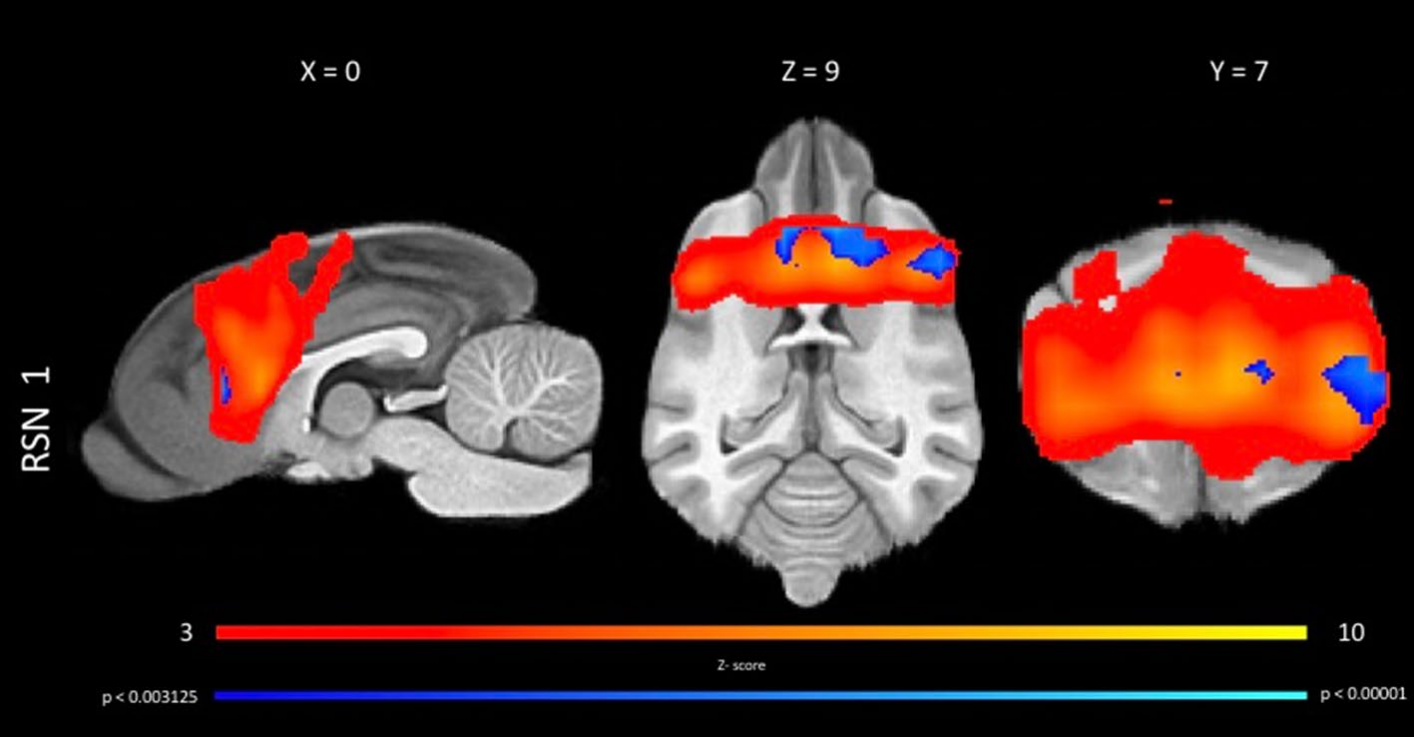
Results of the two-sample *t* tests for increased connectivity in the anterior DMN between the idiopathic epileptic dogs and control dogs in a sagittal, dorsal and transversal plane. The voxel with significantly increased connectivity (p < 0.003125) within the regions of the anterior DMN in epileptic dogs compared to healthy control dogs are shown in blue. The results are overlaid on the local false discovery rate controlled (p < 0.05) thresholded z-maps of the anterior DMN, with red-yellow color encoding using a 3 < Z-score threshold and on a T1W open-source stereotactic atlas^46^. This figure was created using FSLeyes (version 2.1 https://fsl.fmrib.ox.ac.uk/fsl/fslwiki/FSLeyes) and Microsoft Powerpoint (version 16.16.19, http://www.microsoft.com).

Statistical difference of large-scale networks between the epileptic dogs and the healthy controls was found at the rostral component of the DMN and the primary visual network, which showed increased connectivity in the epileptic group relative to healthy controls. In the two-sample *t* test the rostral component of the DMN showed a p-value of 0.00060 (Fig. 3) and the primary visual network a p-value of 0.0018 (taking in account the Bonferroni correction, p had to < 0.003125 for statistical significance).

In the remaining six RSN, no statistical difference between epileptic and controls were found (Table 3).

**Table 3:** Dual regression analysis of independent components for comparison between healthy and epileptic dogs.

Network	Label	Healthy controls—epileptic dogs	Epileptic dogs—healthy controls
Anterior DMN	1	0.019	0.00060*
Posterior DMN	2	0.1174	0.1092
Primary visual	3	0.47	0.0018*
Higher order visual	4	0.6532	0.4502
Higher order visual	5	0.2310	0.2482
Auditory	6	0.9980	0.1784
Auditory	7	0.086	0.6694
Somatosensory	8	0.4260	0.0354

*Significance set at p < 0.003125 after Bonferroni correction.

## Discussion

Significant differences in large-scale networks in epileptic dogs compared to healthy control dogs were found in this study. The most significant difference was identified in the anterior component of the putative DMN using group independent component analysis, a purely data driven approach, and a conservative statistical approach using a Bonferroni correction for significance level. These findings notably support the hypothesis of altered large-scale networks in dogs with natural occurring idiopathic epilepsy.

In human epilepsy alteration detected in rs-fMRI depend on the underlying epileptic syndrome and therefore pattern of rs-fMRI changes have been suggested as potential diagnostic and prognostic biomarker^16^. Canine IE is not a single disease but rather an umbrella term for different epileptic syndromes^60^. These canine epileptic syndromes currently lack stringent standardization^60^ and a therefore a clear homology to a specific human epileptic syndrome is still to be elucidated. In order to standardize the population in absence of well-defined epileptic syndromes, two breeds were chosen, Border Collies and Greater Swiss Mountain Dogs, with assumed genetic background and similar epileptic syndrome in regard to seizure onset, seizure semiology and response to treatment. Only dogs were included that showed the breed specific seizure pattern^26–28^. In both breeds focal onset with secondary generalization is the most common seizure type. In addition, medically refractory epilepsy is common in both breeds^26–28^. The detected increased DMN connectivity in epileptic Border Collies and Greater Swiss Mountain dogs suffering from generalized tonic–clonic seizures suggests a potential target area for further studies, but the degree of functional reorganization in dogs affected by natural occurring idiopathic epilepsy is still unknown and whether or not distinct pattern of altered connectivity, corresponding to specific pattern in human epileptic syndromes, can be found for example in different breed specific epileptic syndrome has to be proven in the future.

Group level ICA as purely data driven whole brain approach was chosen to investigate the RSN in our canine population, as this approach is beneficial in absence of strong assumptions beforehand^61^. Subject specific special maps obtained by dual regression were entered into group level analysis^58^. The individual maps from both groups were analyzed using a GLM-based group level statistical approach. The result of this group level analysis was a map in which the value of each voxel represents the test statistic or p-value obtained (Fig. 3). The highlighted voxel in the result maps (Fig. 3) essentially participated more in the anterior DMN processes of idiopathic epileptic dogs than to those of healthy control dogs. These results reflect differences in both activity and spatial spread of the processes between the groups^57^. In summary, our approach allows us to identify with a high degree of certainty the network as being different in the two groups but does not allow us to draw very firm conclusions about connectivity and shape changes and about the pathophysiological nature of these differences detected.

The DMN is the most extensively studied network in the healthy and diseased brain^62^ and was the first RSN identified in dogs^40^. Compared to humans the DMN in dogs has been reported as dissociated in an anterior and posterior component. This dissociation is also supported by reduced structural connectivity between anterior and posterior cingulate cortex in dogs compared to humans^63^. In the present study, these previously described dissociation could be reproduced^39,40,63^. Nevertheless the DMN in dogs is not as well studied as in humans and proof of the function of what has been identified as DMN in dogs must still be provided^38,39^.

Interestingly, in one of these putative DMN components (anterior DMN) significant differences between idiopathic epileptic dogs and healthy control dogs were found.

Group differences in the DMN in epileptic dogs are of special interest for translational research, as extensive evidence of altered connectivity of the DMN exists in humans affected by epilepsy^15,64^. With few exceptions, the DMN connectivity changes in human epilepsy are reported to be decreased^5,65^. However, increased connectivity within the anterior DMN has been identified in frontal lobe epilepsy alongside with decreased connectivity in humans^65^. Increased functional connectivity was suspected to be caused by compensatory mechanisms in this study. In general, loss of functional integrity accounts for decreased functional connectivity in rs-fMRI^9^, while increased functional connectivity is more difficult to explain. Gorges et al.^9^ hypothesized that increased functional connectivity reflects first/early stage of neurodegenerative diseases and epilepsy, when neuronal reserve is exhausted while normal behavior is still maintained and afterwards it transfers to a state of reduced connectivity when neuronal damage continues. A further explanation could be the loss of inhibitory influence^66^. For these reasons, a possible explanation for the increased functional connectivity seen in the anterior DMN of the epileptic dog population may be due to compensatory mechanisms.

Paralleling the results reported in human epilepsy studies, changes in functional connectivity and network topology are variable in rodent epilepsy models as well^67^. The high connectivity within the DMN in epileptic dogs appears to be similar to results of a kainite-acid rodent model for temporal lobe epilepsy^68^. In this kainic acid epilepsy model increased connectivity within the DMN, specifically the anterior cingulate cortex, without concurrently decreased connectivity in other RSN was found in rs-fMRI under isoflurane anaesthesia^68^. While behavioral, electroencephalographical and neuropathological features of kainate-induced model resemble those of temporal lobe epilepsy in humans, dispersity between rs-fMRI in the rodent model and human temporal lobe epilepsy is a known phenomenon^68^. Increased functional connectivity within the DMN in the rodent kainate-model were thought to be caused by functional reorganization following the induced status epilepticus and an expansion of the multi-synaptic projections within the areas that manifested seizure activity. The reason for this dispersity between human temporal lobe epilepsy and the rodent kainate-model was thought to be caused by bilateral, instead of unilateral, seizure onset in rodents compared to humans, as well as factors including cerebral function reserve, etiology and duration of epilepsy.

Comorbidities are increasingly recognized in canine epilepsy^29,30,33,34^. Changes in RSN concurrent with anxiety and other behavioral comorbidities have been investigated in humans^19^. In people affected by anxiety decreased and increased activation of the anterior DMN^69^ in the region of the anterior cingulate cortex^70–72^ have been found, consistent with impaired function of the DMN during regulation of emotions^73^. Although dog behavior suggestive for anxiety and post ictal anxiety-related aggression were reported by some owners of the epileptic dog population, whether or not the resting state changes detected in the anterior cingulate cortex in the present study also contribute to the behavior changes reported by some owner remains open. However, the fact that dogs have been successfully used as a model of selected human mental and behavioral disorders^74^ and rs-fMRI may also contribute to understand the role in behavioral disorder in canine versus human epilepsy in the future.

All our dogs were scanned in general anaesthesia using an anaesthesia protocol designed to minimize effect on the RSN^39^ and to match the needs for anaesthesia in clinical veterinary patients. More numerous critical parameters were recorded than in other studies during the scan^75^. No significant differences of anaesthesia related parameters were detected between the healthy controls and the idiopathic epileptic patients, indicating no anaesthesia related difference in the RSN between the two groups. Nevertheless, anaesthetic agents, unequally affect RSN and higher-order networks linked to cognition are more severely affected than lower-order, such as basic sensorimotor networks^76^. It is therefore possible, that detection of group differences in higher order networks could have been missed, as they were masked by the effect of general anaesthesia. The DMN can be reliably identified under general anaesthesia. Anterior and posterior hubs of the DMN showed decreased connectivity with continuation of anaesthesia in humans^77^. Effects of different anaesthesia protocols on the DMN of dogs has not been investigated so far. While dissociated anterior and posterior DMN components were detected in awake dogs^40^ and in dogs under Ketamine/Xylazine^40^ as well as under Sevoflurane anaesthesia^39^, this dissociation could not be detected in another study in awake dogs^38^. Because of the standardized anaesthetic protocol applied to both groups, it is less likely that anaesthesia related cofounders cause the changes in the DMN.

Correlation between reduced connectivity and duration of epilepsy has been shown in humans^13^. Eight dogs included in our study had epilepsy for less than 3 month and only 3 dogs suffered from epilepsy for more than 1 year. Dogs affected by seizures have better chance of receiving a clinical-neurological and MRI examination as part of their initial evaluation. It is more difficult to convince owners of follow-up examinations. It remains unclear if, by performing long-term follow up rs-fMRI in the epileptic dogs, disease progression leads to an increased number of networks showing differences in comparison to controls and if reduced instead of increased connectivity can be found with longer diseases duration.

In rodent models increased connectivity appears to be triggered by status epilepticus via functional reorganization and an expansion of the multi-synaptic projections^68^. However, only one of the included dogs had experienced status epilepticus before the MRI scan. Therefore, it is unlikely that the increased connectivity within the epileptic dog group is triggered by status epilepticus.

There are several limitations to the study. First of all, the number of dogs was limited due to ethical reasons of animal welfare for the healthy controls and due to restricted availability of clinical patients. No statistical differences between groups could be identified regarding to age, weight and anaesthesia related parameters, but it was not possible to match for age, sex and environmental background with the number of animals included. The same holds true for distribution between breeds: more border collies were included in the epileptic dogs group compared to the healthy controls, but the lack of significant differences in any of the detected RSN between the breeds in healthy controls confirms that RSN are comparable between breeds.

Second, the onset of the seizures was not documented in more than half of the dogs, and the seizure category remains unknown. In a clinical setup, this is a challenging event to document, since the majority of dog owners witness first the generalized ictal episode or post-ictal phase first.

Third, electroencephalography was not available immediately before or during the scan to rule out inter-ictal spike activity. However, non-invasive scalp electroencephalography has several issues in dogs compared to humans related to skull thickness, muscle artefacts and restricted number of electrodes limiting its use in clinical studies^24^.

Fourth, while pre- and postprocessing of rs-fMRI has been further developed over the last few years, rs-fMRI in animals, especially dogs is still in its infancy^78^. Software for registration is optimized for human use and how, for example, the artefacts in the area of the frontal sinus can be minimized by optimizing the scanning parameters is still open. Therefore, we have to treat the results with some caution, especially since the artefact of the frontal sinus, which is much more pronounced in dogs than in humans (Fig. S2), is directly adjacent to the anterior DMN. It is possible that parts of the network are obscured by signal extinction in the area of the artefact. This caution must also include the interpretation of the lateralization of the area of increased connectivity visible in the anterior DMN (Fig. 3). The apparent lateralization might be influenced by differences in signal to noise ratio between groups in this area (further information can be found in the Supplementary M1 and the Supplementary Fig. S4).

Last, all idiopathic epileptic dogs would have been investigated while drug-naïve to test the effects of epilepsy alone without the bias of medication, ideally. However, for ethical reasons it was not possible to withhold medications in a clinical scenario. More than 50% of the idiopathic epileptic dogs (9/17) were treated with various drug combinations compared to none in the healthy control group. Unfortunately, assessing their individual effect statistically in this small group of patients was not possible.

The present study further confirms the feasibility to perform rs-fMRI in dogs in a clinical setting and indicates differences between healthy controls and epileptic dogs, and consequently enhancing the potential of canine epilepsy as a translational model for human epileptic syndromes. To allow comparison of data across multiple MRI scans from different animals of a given species, it is essential to align the acquired images into a common anatomical space. We used a recently published canine neuroimaging atlas^46^ that allows reporting of fMRI results with standard coordinates and in relation to anatomical structures.

## Conclusion

Increased functional connectivity in large-scale brain networks, the anterior DMN and the primary visual network, can be found in Border Collies and Greater Swiss Mountain Dogs affected by natural occurring canine idiopathic epilepsy compared to healthy control dogs. This group level differences between epileptic dogs and healthy control dogs identified with using a rather simple data driven approach could serve as a starting point for more advanced resting state network analysis in epileptic dogs.

## Supplementary Information

Supplementary Figure S1.

Supplementary Figure S2.

Supplementary Figure S3.

Supplementary Figure S4.

Supplementary Legends.

Supplementary Information.

## Data Availability

The raw imaging data generated during the current study are available on https://openneuro.org/datasets/ds003830. The datasets generated and analyzed during the current study are available from the corresponding author on reasonable request.

## Abbreviations

BOLD: Blood oxygenation level dependent
DMN: Default mode network
EPI: Gradient-echo planar imaging
EtCO_2_: End-tidal partial pressure of carbon dioxide
FOV: Field of view
HR: Heart rate
ICA: Independent component analysis
IE: Idiopathic epilepsy
MAP: Mean arterial blood pressure
RR: Respiratory rate
rs-fMRI: Resting state functional magnetic resonance imaging
RSN: Resting state network
SpO_2_: Percutaneous arterial oxygen saturation
