# Supplementary figures and images for: Increased resting state connectivity in the anterior default mode network of idiopathic epileptic dogs
Source: Sci Rep. 2021 Dec 13;11:23854. doi: 10.1038/s41598-021-03349-x (PMC8668945; doi:10.1038/s41598-021-03349-x)

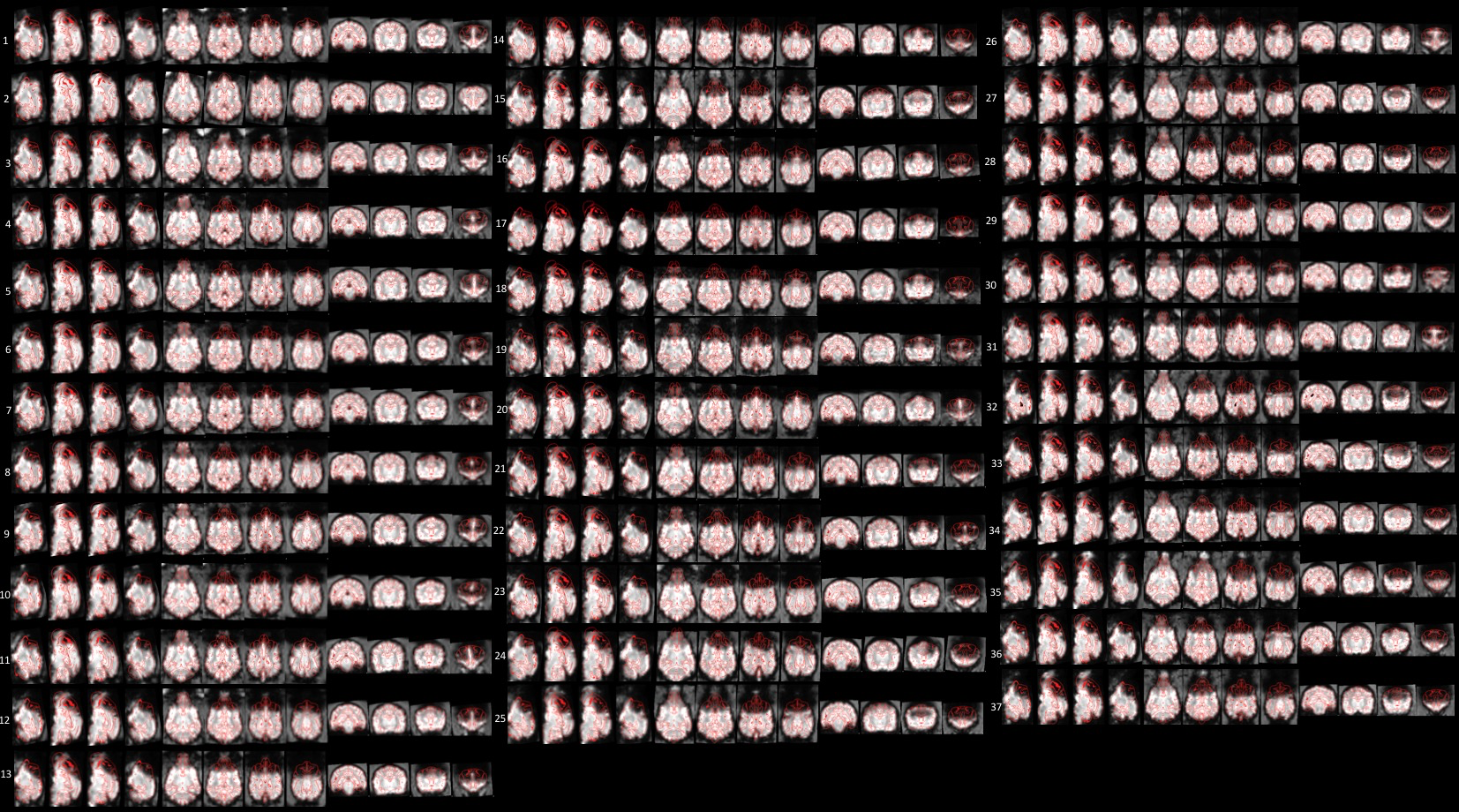

Supplement: Supplementary file 1 — Supplementary Figure S1. [file 41598_2021_3349_MOESM1_ESM.tiff]

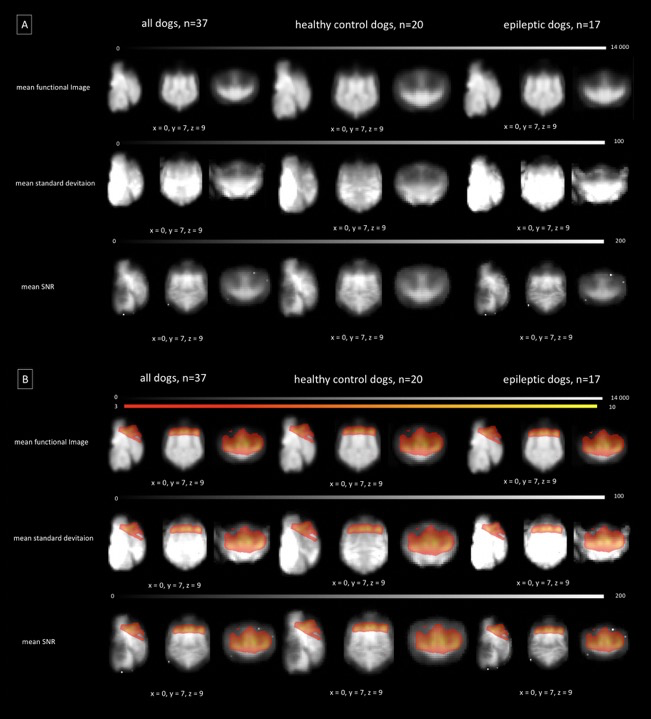

Supplement: Supplementary file 2 — Supplementary Figure S2. [file 41598_2021_3349_MOESM2_ESM.tiff]

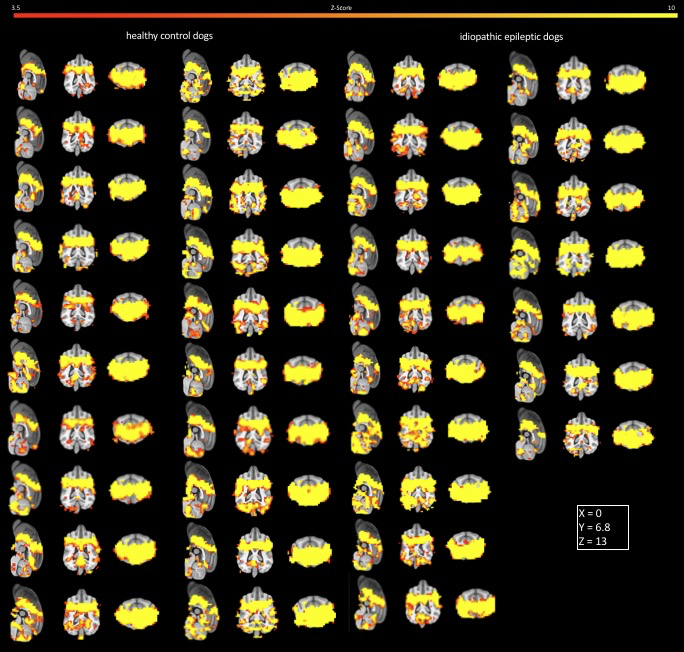

Supplement: Supplementary file 3 — Supplementary Figure S3. [file 41598_2021_3349_MOESM3_ESM.tiff]

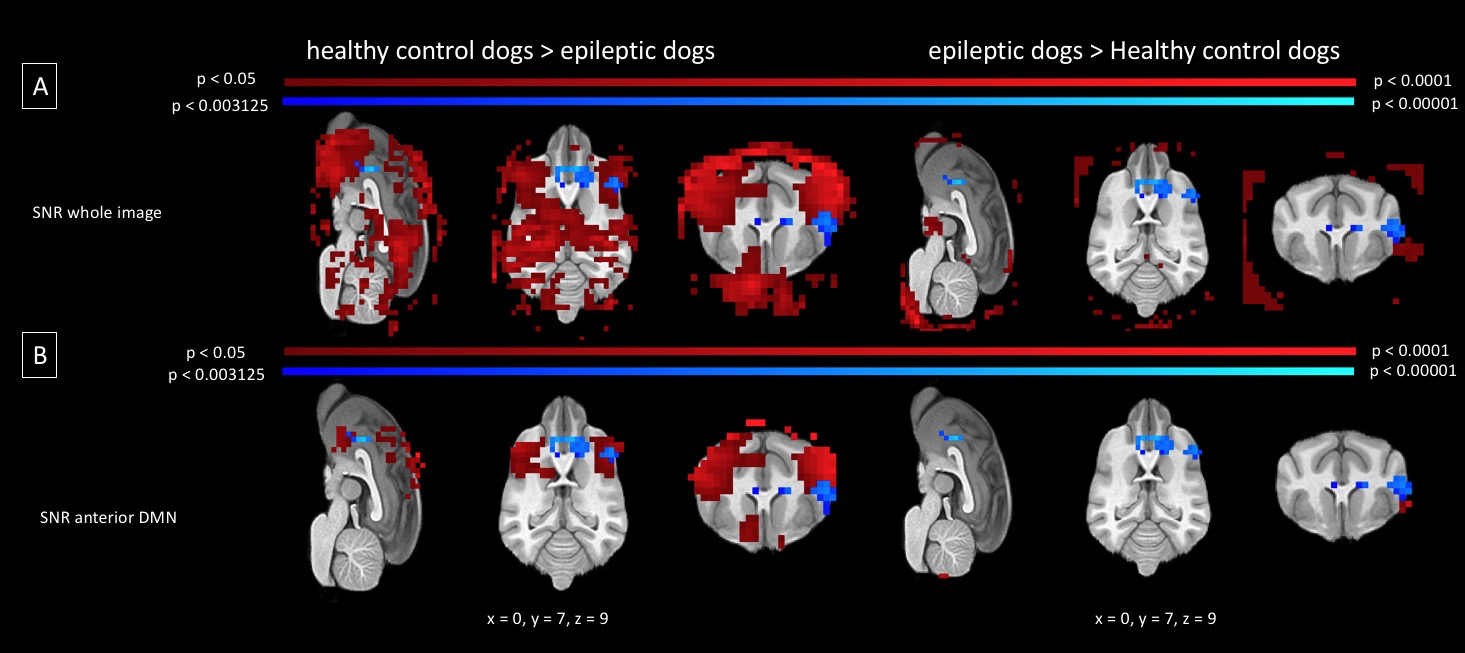

Supplement: Supplementary file 4 — Supplementary Figure S4. [file 41598_2021_3349_MOESM4_ESM.tiff]
