# Supplementary material for: Increased resting state connectivity in the anterior default mode network of idiopathic epileptic dogs
Source: Sci Rep. 2021 Dec 13;11:23854. doi: 10.1038/s41598-021-03349-x (PMC8668945; doi:10.1038/s41598-021-03349-x)
Supplement: Supplementary file 5 — Supplementary Legends. [file 41598_2021_3349_MOESM5_ESM.docx]

Figure S1: Summery of the registration functional images to T1W open-source stereotactic atlas[46] of each of the 37 dogs included in the study. This figure was created using FSLeyes (version 2.1 https://fsl.fmrib.ox.ac.uk/fsl/fslwiki/FSLeyes) and Microsoft Powerpoint (version 16.16.19. www.microsoft.com).

Figure S2: Sagittal, dorsal and transversal mean functional images, mean standard deviation and SNR of all dogs, healthy control dogs and epileptic dogs are shown alone (A) and overlaid with the anterior DMN (B). This figure was created using FSLeyes (version 2.1 https://fsl.fmrib.ox.ac.uk/fsl/fslwiki/FSLeyes) and Microsoft Powerpoint (version 16.16.19. www.microsoft.com).

Figure S3: Sagittal, dorsal and transversal images per subject of the spatial maps estimated in stage two of the dual regression. This figure was created using FSLeyes (version 2.1 https://fsl.fmrib.ox.ac.uk/fsl/fslwiki/FSLeyes) and Microsoft Powerpoint (version 16.16.19. www.microsoft.com).

Figure S4: Results of the two-sample t-test for the SNR of whole image are shown overlaid on the T1W open-source stereotactic atlas[46] (A), as well as the results of the two-sample t-test only comparing the area of the anterior DMN overlaid on the T1W open-source stereotactic atlas[46]: For further comparison the results of the dual regression for the this network are also shown (B). The voxel with a significant increase (p < 0.05) in SNR in the two-sample t-test are highlighted in red and voxel with significant increased connectivity (p < 0.003125) in the epileptic dogs compared to the healthy control dogs are highlighted in blue. This figure was created using FSLeyes (version 2.1 https://fsl.fmrib.ox.ac.uk/fsl/fslwiki/FSLeyes) and Microsoft Powerpoint (version 16.16.19. www.microsoft.com).
