# Supplementary material for: Increased resting state connectivity in the anterior default mode network of idiopathic epileptic dogs
Source: Sci Rep. 2021 Dec 13;11:23854. doi: 10.1038/s41598-021-03349-x (PMC8668945; doi:10.1038/s41598-021-03349-x)
Supplement: Supplementary file 6 — Supplementary Information. [file 41598_2021_3349_MOESM6_ESM.pdf]

Supplementary Material: M1

Calculation of signal to noise ratios and comparison of signal to noise ratios between groups

Materials and Methods:

The SNR for every dog was calculated using fslmaths, part of FSL (FMRIB's Software Library v6.0, www.fmrib.ox.ac.uk/fsl). The mean functional images and the standard deviation from each dog was calculated from the fMRI series registered to standard space of each dogs. The SNR of each dog was calculated by dividing these mean functional images by the calculated standard deviations of each dog.
Mean functional images per group were calculated by first creating a 4D file of all mean functional images per group using fslmerge, part of FSL (FMRIB's Software Library v6.0, www.fmrib.ox.ac.uk/fsl), and then by calculating the mean image of the group 4D file. The same procedure was performed for the standard deviation maps and the SNR maps.
To compare the SNR between the epileptic dogs and the healthy control dogs a two-sample t-test was performed using FSL's randomize permutation-testing tool (5000 permutations)[1]. The two-sample t-test was performed without mask image and with the sDMN as mask image. Voxel with increased or decreased SNR were confirmed using a significance level of $p<0.05$.

Results:
The results of the mean functional images per group, the mean standard deviation per group as well the mean SNR per group are shown in supplementary figure S2a. The results overlaid with the anterior DMN are shown in supplementary figure S2b.
The results of the two-sample t-test are shown in supplementary figure S4. The test without mask image is shown in figure S3a, the results of the two-sample t-test with mask image (anterior DMN) is shown in figure S3b. The results are overlaid with the T1 atlas images and the voxels with statistically significantly increased connectivity in the epileptic dog group (dual regression results).

1 Winkler, A. M., Ridgway, G. R., Webster, M. A., Smith, S. M. & Nichols, T. E. Permutation inference for the general linear model. *NeuroImage* **92**, 381-397, doi:10.1016/j.neuroimage.2014.01.060 (2014).
